# Beyond Fixed-Size Skyrmions in Nanodots: Switchable Multistability with Ferromagnetic Rings

**DOI:** 10.1021/acs.nanolett.5c02678

**Published:** 2025-09-11

**Authors:** Mateusz Zelent, Maciej Krawczyk, Konstantin Y. Guslienko

**Affiliations:** † Faculty of Physics and Astronomy, 49562 Adam Mickiewicz University in Poznan, Uniwersytetu Poznańskiego 2, PL-61-614 Poznan, Poland; ‡ Faculty of Physics and Astronomy, Adam Mickiewicz University, Poznan, Uniwersytetu Poznańskiego 2, PL-61-614 Poznan, Poland; ¶ Depto. Polimeros y Materiales Avanzados: Fisica, Quimica y Tecnologia, Universidad del País Vasco, UPV/EHU, 20018 San Sebastian, Spain; § EHU Quantum Center, University of the Basque Country, UPV/EHU, 48940 Leioa, Spain; ∥ IKERBASQUE, the Basque Foundation for Science, 48013 Bilbao, Spain

**Keywords:** magnetic memory devices, spin-based logic, neuromorphic computing architectures

## Abstract

We demonstrate a novel approach to controlling and stabilizing magnetic skyrmions in ultrathin multilayer nanostructures through spatially engineered magnetostatic fields generated by ferromagnetic nanorings. Using analytical modeling and micromagnetic simulations, we show that the stray fields from a Co/Pd ferromagnetic ring with out-of-plane magnetic anisotropy significantly enhance the Néel-type skyrmion stability in an Ir/Co/Pt nanodot, even stabilizing the skyrmion in the absence of Dzyaloshinskii–Moriya interactions. We demonstrate precise control over the skyrmion size and stability. We observe a multistability phenomenon, where the skyrmion can be stabilized at two or more distinct equilibrium diameters depending on the ring’s magnetization orientation. These stable states exhibit energy barriers that substantially exceed thermal fluctuations at room temperature, suggesting potential applications in robust multibit memory storage. Furthermore, we demonstrate that a skyrmion can be switched between two metastable states using a suitably designed nanosecond magnetic field pulse. Our findings pave the way for advanced spintronic nanodevices.

## Graphical abstract

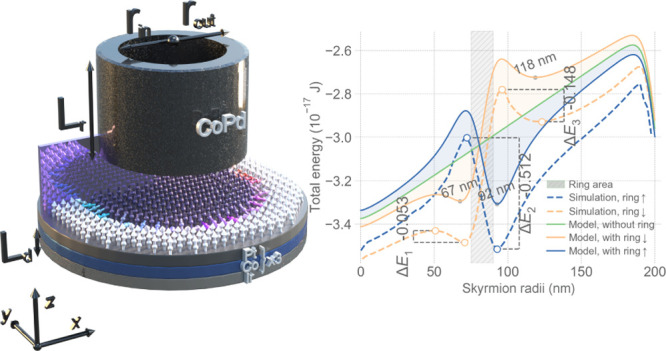

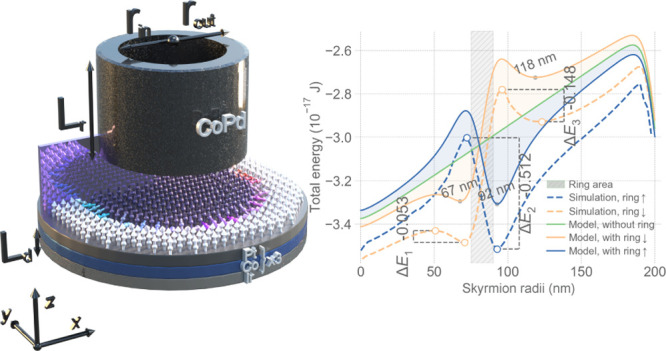

Magnetic skyrmions, nanoscale swirling spin textures,^1^ can be stabilized at room temperature in chiral magnets and magnetic multilayer films exhibiting strong interfacial Dzyaloshinskii–Moriya interactions (DMI).^2^ These particle-like spin configurations display ultralow critical currents for motion, rendering them promising information carriers for high-density devices such as the proposed racetrack memories and logic gates, wherein data are encoded by the presence or absence of individual skyrmions.^2,3^ The combination of nanometer-scale dimensions, topological protection, and efficient electrical manipulability positions skyrmions as attractive building blocks for next-generation spintronic memory and computing technologies.^4–6^

However, a significant challenge for practical skyrmion-based devices lies in ensuring their robust stability under ambient field-free conditions. In many known skyrmion-hosting materials, skyrmions are stable only within a narrow range of low temperatures or require bias external magnetic fields.^7,8^ Isolated skyrmions in single-layer films are often metastable, prone to collapse or elongate into stripe domains in the absence of a stabilizing field.^9^ This reliance on external magnetic fields complicates device integration and increases the power requirements. Furthermore, conventional skyrmions typically possess a single equilibrium size determined by material parameters,^10,11^ offering only a binary state (presence or absence of a skyrmion) for information storage.^12^ While useful, this binary nature limits the stored information density and potential functionality of skyrmion-based devices.

Achieving multiple stable skyrmion configurations (i.e., multistability) within the same nanostructure could enable multilevel memory cells or novel logic states.^13,14^ Such precise control over the skyrmion states has remained elusive. Although skyrmions with higher-order topological winding numbers (multiturn skyrmions) were observed in specific bulk chiral magnets, suggesting the possibility of multistate topological textures, switching between these states is nontrivial.^15^ Consequently, stable multistate skyrmions were not realized in practical device geometries. This gap underscores the need for innovative methods to enhance skyrmion stability and unlock additional stable states for advanced applications.

Recent studies^16–18^ have provided crucial insights into magnetic skyrmions in confined geometries, highlighting the critical role of boundary conditions on skyrmion behavior and stability within ultrathin and multilayer nanodots. While our previous research^19^ demonstrated the feasibility of bistable skyrmion states with two distinct sizes in multilayer nanodots with perpendicular magnetic anisotropy, these states existed only within very narrow ranges of material parameters and were typically separated by an inherently low energy barrier. This limitation resulted in short lifetimes against thermal fluctuations and presented significant challenges for reliable practical applications. Theoretical work^11^ provides a quantitative understanding of how the skyrmion size and stability depend sensitively on a balance of the exchange, anisotropy, DMI, and Zeeman energies, confirming the energetic difficulty in stabilizing compact skyrmion states. Furthermore, Büttner et al.^20^ drew a distinction between skyrmions primarily stabilized by DMI versus those stabilized by magnetic stray fields. Their analysis revealed that while DMI can, in principle, stabilize sub-10 nm skyrmions, achieving this at room temperature and zero magnetic field is extremely difficult in commonly used ferromagnetic multilayers (such as Co-based systems). This often requires alternative materials (e.g., ferrimagnets) or nonzero applied fields.

Another way to control skyrmions is to use magnetostatic fields from neighboring magnetic layers or superconductors.^21,22^ Verba et al.^23^ showed that dipolar coupling with a hard magnetic layer patterned as an antidot could stabilize magnetic vortex states in soft nanodots located under the antidots, significantly extending their stability range. In a recent work,^24^ we explored a hybrid system consisting of a skyrmion-hosting nanodot placed on an in-plane magnetized soft ferromagnetic stripe. We found that the mutual magnetostatic interaction leads to interesting effects: the skyrmion induces a magnetic imprint on the stripe, and the stray field from this imprint, in turn, acts back on the skyrmion. This interaction breaks the skyrmion’s circular symmetry, leading to an asymmetric (egg-shaped) deformation, enhances its stability (allowing stabilization at lower DMI values), increases its overall size compared to an isolated dot, and even introduces skyrmion bistability within a specific DMI range.^24^ This work highlights how the magnetic stray fields in hybrid structures can profoundly modify skyrmion properties and potentially mitigate the skyrmion Hall effect.

Building upon these insights—particularly the potential for manipulating skyrmions via engineered magnetostatic interactions from adjacent layers^23,24^ —we are now investigating nanostructures with a geometry that is specifically designed to enhance the stability of skyrmions and to achieve their multistability. We propose the design of a skyrmion hosting device where a ferromagnetic ring above a multilayer nanodot is designed to achieve multistable skyrmions with enhanced stabilities.

The magnetostatic stray field generated by this ring provides a stabilizing field within the nanodot’s interior, acting as an integrated bias field analogous to an external magnetic field. This approach is particularly effective for stabilizing very small skyrmions (diameters <50 nm) by significantly deepening the skyrmion energy minimum, providing a robust barrier against its collapse, even in zero external field and without DMI. Remarkably, we find that the interplay between the ring’s stray field and the skyrmion leads to multiple stable skyrmion states. Specifically, for appropriate ring dimensions and magnetization, the system can support two distinct stable skyrmion configurations within the dot, a “small-radius” skyrmion and an “expanded” skyrmion, both corresponding to local energy minima. Furthermore, we propose and numerically demonstrate the switching between both skyrmion states by applying short (below 1 ns) magnetic field pulse. The implications of these findings are significant for skyrmion-based spintronics. Enhanced stability ensures reliable information retention against thermal fluctuations and perturbations, crucial for memory and logic applications. The ring-stabilized skyrmion design provides a practical pathway to harness multistable topological states for multilevel memory cells in a simple geometry.

Figure 1(a) illustrates the geometry of our proposed device. It consists of a Co/Pd multilayer ring (outer radius *r*_out_: 65 nm, inner radius *r*_in_: 55 nm, height *L*_r_: 10 nm) with sufficiently high perpendicular magnetic anisotropy (PMA) to ensure saturation. The ring is positioned 1.2 nm above an Ir/Co/Pt multilayer nanodot separated by a nonmagnetic material. The nanodot has a fixed thickness *L*_d_ = 1.2 nm. The nanodot’s radius *r*_d_ and the ring’s thickness and its inner/outer radii serve as tunable parameters in our analysis. Detailed material parameters are provided in the Supporting Information (SI).

**Figure 1:**
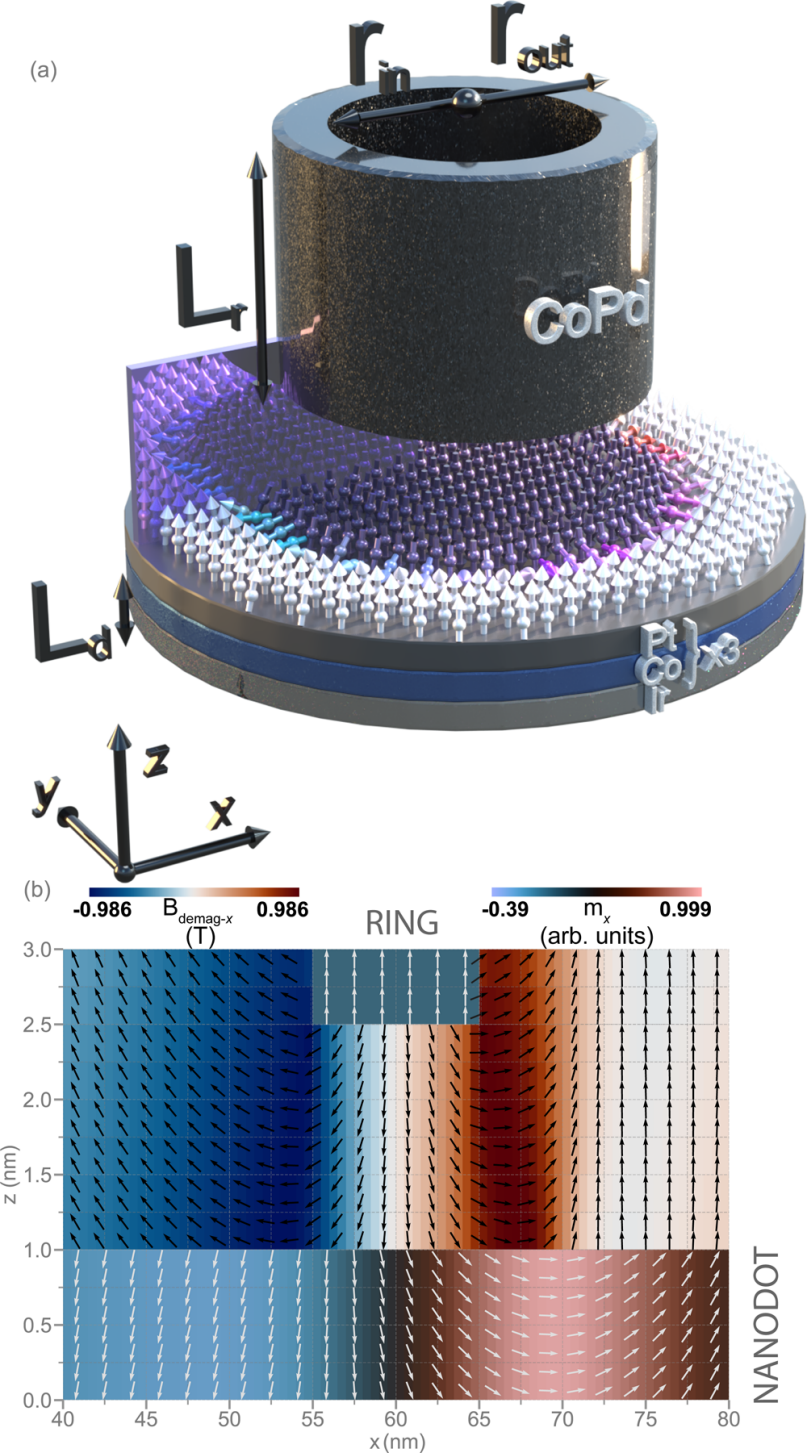
(a) Schematic representation of the proposed device consisting of an Ir/Co/Pt multilayer nanodot with radius *r*_d_ = 100 nm hosting the magnetic skyrmion and a Co/Pd ferromagnetic ring. The purple semitransparent cuboid represents the spatial cross-section at which the magnetization texture and demagnetization field are shown in (b). (b) Visualization of the numerically calculated in-plane component of the demagnetizing field (*B*_demag,*x*_, left color map and vector field *B*_demag,*x−z*_ *,*black arrows) outside of the ferromagnets and the corresponding in-plane magnetization component (*m*_*x*_, right colormap and vector field *m*_*x−z*_, white arrows) inside the nanodot and ring, revealing steep field gradients at the inner and outer ring boundaries that induce pronounced magnetization tilting. Color scale represents field strength, with red/blue indicating positive/negative values. The inner and outer ring radii are *r*_in_ = 55 nm and *r*_out_ = 65 nm, respectively.

In our investigations, we use an in-house version of Mumax3,^25,26^ called AMUmax,^27^ for the micromagnetic simulations, which solve the Landau–Lifshitz–Gilbert equation including magnetostatic, exchange, anisotropy, Zeeman, and DMI fields. To gain deep physical insight into the stability of Néel skyrmions within the ultrathin magnetic nanodot, we developed an analytical model. Details of both methods are presented in the SI.

The spatially nonuniform stray field generated by the ferromagnetic ring [see Figure 1(b) and Figures S1 in the SI] profoundly shapes the skyrmion energy landscape within the nanodot. This influence stems from the interaction between the field and the skyrmion’s Néel domain wall, which preferentially may stabilize the skyrmion in regions where the in-plane stray field component is strong, notably near the inner and outer edges of the ring. Depending on the local field’s alignment with the skyrmion’s chirality, these regions induce effective energy wells or barriers, fundamentally altering the energy profile. This capability for spatial energy landscape engineering allows for precise control over the skyrmion’s equilibrium radius and substantially enhances its stability, particularly for compact skyrmions, by establishing high energy barriers against collapse. Such an engineered energy landscape is pivotal for realizing the observed multistability.

We start the analysis with the reference system, i.e., the nanodot without the ring and zero DMI (solid green curve in Figure 2). Here, the nanodot exhibits no energy minima and thus no stable equilibrium for a skyrmion, confirming inherent skyrmion instability without DMI. Conversely, introducing the ring (*r*_in_ = 75 nm and *r*_out_ = 90 nm, *L*_r_ = 10 nm) results in clear energy minima, effectively stabilizing skyrmions at specific diameters, for both magnetization orientations in the ring (the blue and orange lines). Importantly, the winding number of these skyrmions is −0.99 (see the SI for details), which confirms their topological nature. Both analytical calculations (solid lines) and micromagnetic simulations (dashed lines) consistently confirm this stabilization mechanism and confirm that our theoretical approach accurately captures the essential physics of magnetostatic skyrmion stabilization.

**Figure 2:**
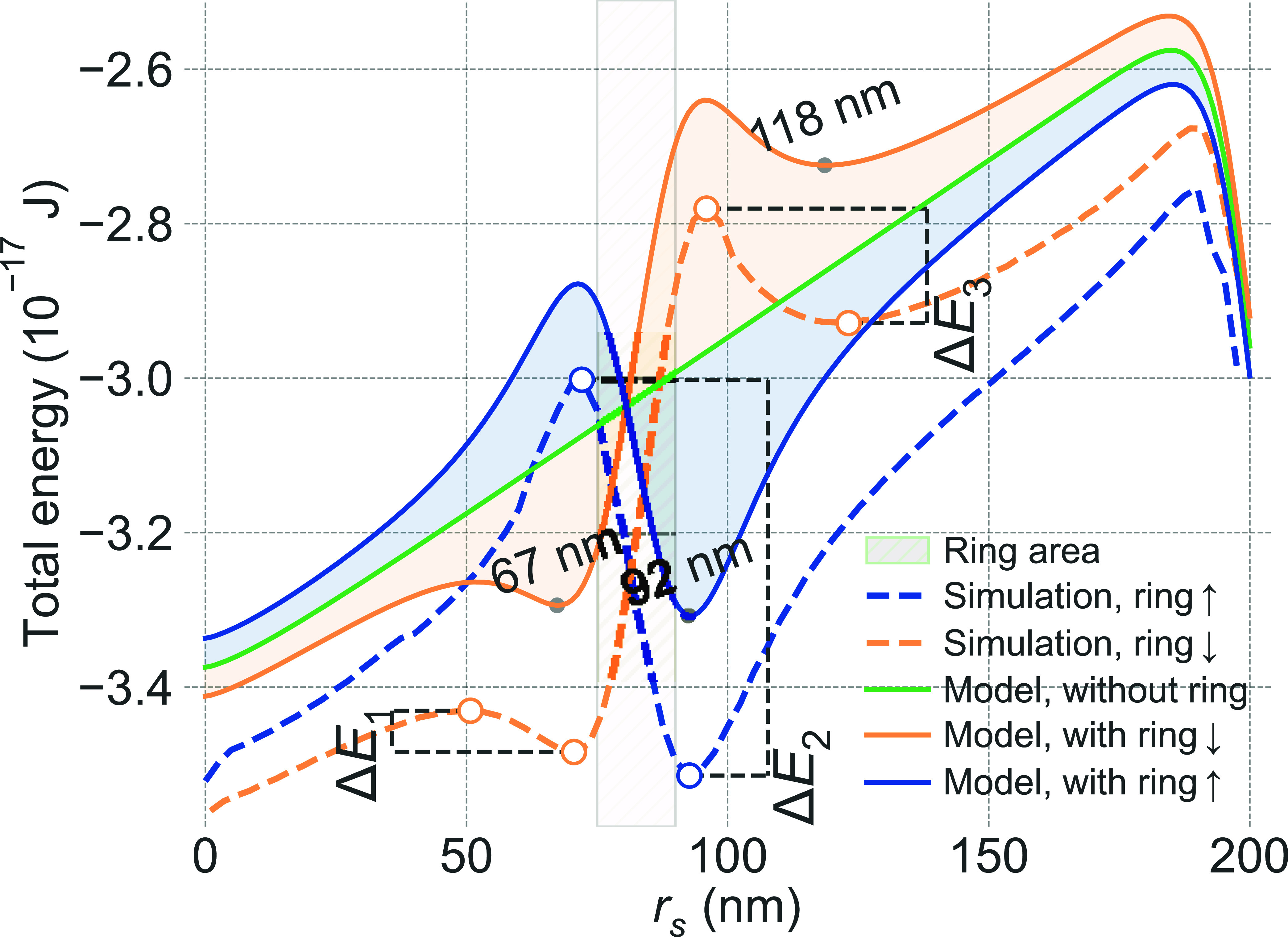
Total magnetic energy of the Néel skyrmion in the DMI-free nanodot as a function of its radius *r*_s_. The shaded region indicates the spatial extent of the ring, with inner and outer radii of *r*_in_ = 75 nm and *r*_out_ = 90 nm, respectively. Solid lines represent the analytical model, while dashed lines correspond to micromagnetic simulations. The green curve denotes the reference case without the ring for a nanodot of thickness *L*_d_ = 1.2 and radius *r*_d_ = 200 nm. The orange and blue curves correspond to cases where the ring is magnetized downward (*↓*) and upward (*↑*), respectively.

While simplifications in the analytical model lead to a consistent offset in the absolute magnetic energy values compared to micromagnetic simulations, likely due to under-representation of slight edge effects in confined nanodots (see Figure 2 and Figure S1 in the SI), the excellent agreement in the energy landscape shape and the prediction of stable skyrmion sizes (position of minima) is evident. Thus, Figure 2 demonstrates a remarkable finding: a Néel skyrmion is stabilized purely by the magnetostatic stray field from the ring in the absence of DMI in the nanodot, with the model correctly predicting the stable states.

The skyrmion stabilization mechanism is strongly dependent on the relative orientation of the ring magnetization and the skyrmion’s chirality and how the resulting ring stray field interacts with the skyrmion’s Néel domain wall. Considering the case of upward ring magnetization (*↑*, blue curves), as shown in Figure 1(b) and Figure S2 in the SI, outside the ring, particularly near its outer edge, the in-plane component of this stray field has a rotational profile that aligns favorably with the radial rotation of the skyrmion’s magnetization. This alignment contributes negatively to the total energy, creating a potential well that stabilizes the Néel skyrmion at a larger radius (around *r*_s_ ≈ 93 nm), where its domain wall coincides with this region of aligned field (see Figure S5). Conversely, at radii where the ring field opposes the domain wall rotation, an energy barrier is formed. The resulting deep energy well at this radius yields an exceptional stability barrier (Δ*E*_2_ = 0.512 × 10^–17^ *J*, Δ*E*_2_/*k*_B_ *T* ≈ 1249), sufficient for protection against thermal fluctuations (see SI Table 1 with discussion of all energy barriers Δ*E*_1_ – Δ*E*_3_) with skyrmion thermal lifetimes).

In contrast, for downward ring magnetization (*↓*, orange curves), the stray field profile is effectively inverted. The in-plane field now opposes the skyrmion’s domain-wall rotation in certain regions, particularly outside the ring, creating an energy barrier for larger skyrmions. This results in two distinct minima of the skyrmion energy at approximately 67 and 118 nm. The stabilization of the inner skyrmion (*r*_s_ ≈ 67 nm) is determined by the field inside the ring, while the outer skyrmion (*r*_s_ ≈ 118 nm) is stabilized near the outer edge of the ring, where field alignments still offer energy minima despite the overall opposing field direction relative to the core magnetization. Despite these different stabilization processes, the depths of these minima—and thus their thermal stabilities—are comparable, underscoring the robustness of magnetostatic stabilization. Having established that a skyrmion can be stabilized in a nanodot solely through magnetostatic interactions with the ring, we next examine the more general case where both DMI and the ring stray field contribute simultaneously. This scenario has significant practical relevance for spintronic applications, as most heavy-metal–ferromagnet interfaces naturally exhibit some degree of DMI. Figure 3 presents the main effects of introducing DMI in combination with the ring-induced magnetostatic field. The calculations were performed for a nanodot (thickness, *L*_d_ = 1.2 nm) with two different DMI strengths: *D* = 1.8 mJ/m^2^ (panel a) and *D* = 2.6 mJ/m^2^ (b). The shaded regions indicate the spatial extent of the ring, with inner and outer radii varying between the two configurations: (a) *r*_in_ = 85 nm, *r*_out_ = 100 nm and (b) *r*_in_ = 55 nm, *r*_out_ = 70 nm.

**Figure 3:**
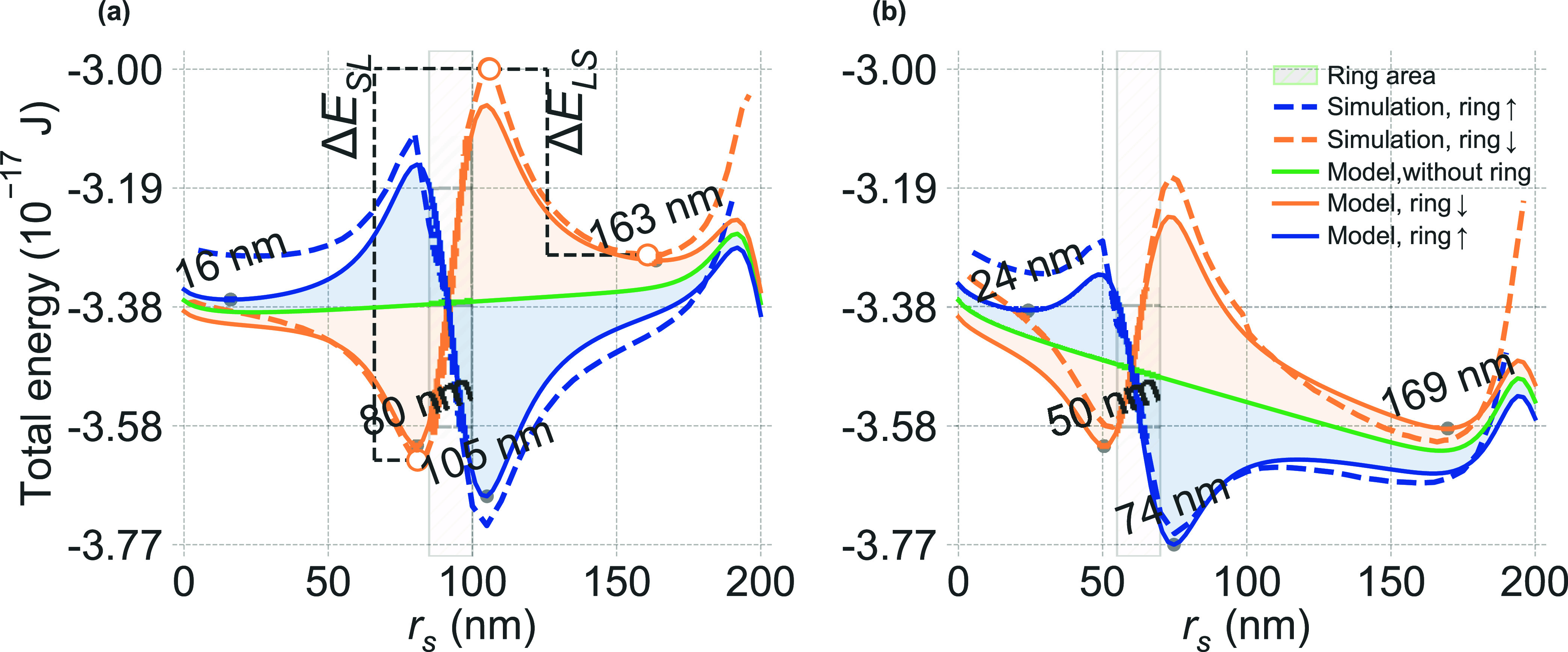
Total energy of the Néel skyrmion as a function of its radius *r*_s_, comparing analytical model predictions (solid lines) with micromagnetic simulations (dashed lines) for different ring polarization and DMI strengths for the nanodot radius *r*_d_ = 200 nm. Gray dots indicate energy minima, with corresponding approximate skyrmion radii labeled. The shaded gray region marks the radial extent of the ring. The green curve denotes the reference case, the nanodot without the ring. (a) DMI strength *D* = 1.8 mJ/m^2^,^28^ with ring dimensions *r*_in_ = 85 nm, *r*_out_ = 100 nm. For the ring magnetized upward (*↑*, blue lines), minima appear at *r*_s_ ≈ 16 nm and *r*_s_ ≈ 105 nm. For the ring magnetized downward (*↓*, orange lines), minima are observed at *r*_s_ ≈ 80 nm and *r*_s_ ≈ 163 nm. (b) DMI strength *D* = 2.6 mJ/m^2^,^29^ with ring dimensions *r*_in_ = 55 nm, *r*_out_ = 70 nm. For the ring magnetized upward (*↑*, blue lines), minima appear at *r*_s_ ≈ 24 nm, *r*_s_ ≈ 74 and *r*_s_ ≈ 169 nm, and for the ring magnetized downward (*↓*, orange lines), a minimum is observed at *r*_s_ ≈ 50 nm, with another minimum at *r*_s_ ≈ 169 nm.

In Figure 3(a), we observe distinct energy landscapes for different ring polarization states for a nanodot with a lower *D*. For downward (*↓*) ring magnetization (orange curve), the energy features two distinct minima at skyrmion radii of approximately 80 nm (smaller skyrmion, S) and 163 nm (larger skyrmion, L). With upward (*↑*) ring magnetization (blue curve), the energy also reveals two stable skyrmions: a smaller skyrmion in a shallow minimum at approximately 16 nm and a larger skyrmion in a deeper minimum at approximately 105 nm. The energy barrier for transitioning from the smaller skyrmion to the larger one (Δ*E*_S→L_) is approximately 0.65 × 10^–17^ J. The barrier for the reverse transition, from the larger skyrmion state back to the smaller one (Δ*E*_L→S_), is lower, at approximately 0.29 × 10^–17^ J; however, both are well above the thermal energy at room temperature (*k*_B_ *T* ≈ 4.14 × 10^–21^ J at 300 K), ensuring thermal stability. Both these interstate barriers, while differing in magnitude, indicate stabilities for the smaller and larger skyrmion configurations in this upward ring polarization. Importantly, for the reference sample, i.e., a nanodot without the ring (green line), we observe only a single shallow minimum at approximately 16 nm, corresponding to a smaller skyrmion state.

Figure 3(b) demonstrates how increasing the DMI strength to *D* = 2.6 mJ/m^2^, with the ring dimensions adjusted, modifies the energy landscape. With downward ring polarization, stable skyrmions appear at 50 and 169 nm. For upward polarization, we observe three stable states at 24, 74, and 169 nm, demonstrating clear multistability. The reference case without the ring (green curve) exhibits only a single minimum at 169 nm, highlighting how the ring-induced magnetostatic field fundamentally transforms the skyrmion stability energy landscape.

These results demonstrate that the interplay between DMI and the spatially varying magnetostatic field from the ring creates complex energy landscapes, which can be designed to possess multiple well-defined minima for stable skyrmions. The positions and depths of these minima—and consequently the stable skyrmion diameters—can be precisely engineered by controlling the ring dimensions and its magnetization direction as well as the DMI of the nanodot. This remarkable multistability emerges from the competition between the chirality imposed by the DMI and the effective chirality induced by the magnetostatic field from the ring. When these chiralities align, existing energy minima deepen and new metastable states may emerge. Conversely, when they oppose each other, certain stability points are suppressed while others are enhanced. Having established the existence of multistable skyrmion states in a nanodot, we now address the critical question of how to reliably switch between these states in a controlled manner—a prerequisite for practical memory and logic applications. For this demonstration, we select the energy landscape shown in Figure 4(a), featuring multiple local minima for a system characterized by the following parameters: *D* = 1.2 mJ m^–2^, *L*_d_= 1.2 nm, ring inner radius *r*_in_ = 40 nm, outer radius *r*_out_ = 60 nm, height *L*_r_ = 3.6 nm, and ring magnetization oriented downward. The identified stable states correspond to skyrmion radii of *r*_s_ ≈ 36 nm and *r*_s_ ≈ 84 nm. These states are separated by direction-dependent energy barriers: the barrier for transitioning from the smaller to the larger skyrmion state, Δ*E*_S→L_, is approximately 0.25 × 10^–17^ J, while the barrier for the reverse transition, Δ*E*_L→S_, is approximately 0.5 × 10^–18^ J. This configuration creates a pathway for deterministic switching between skyrmions of different diameters.

**Figure 4:**
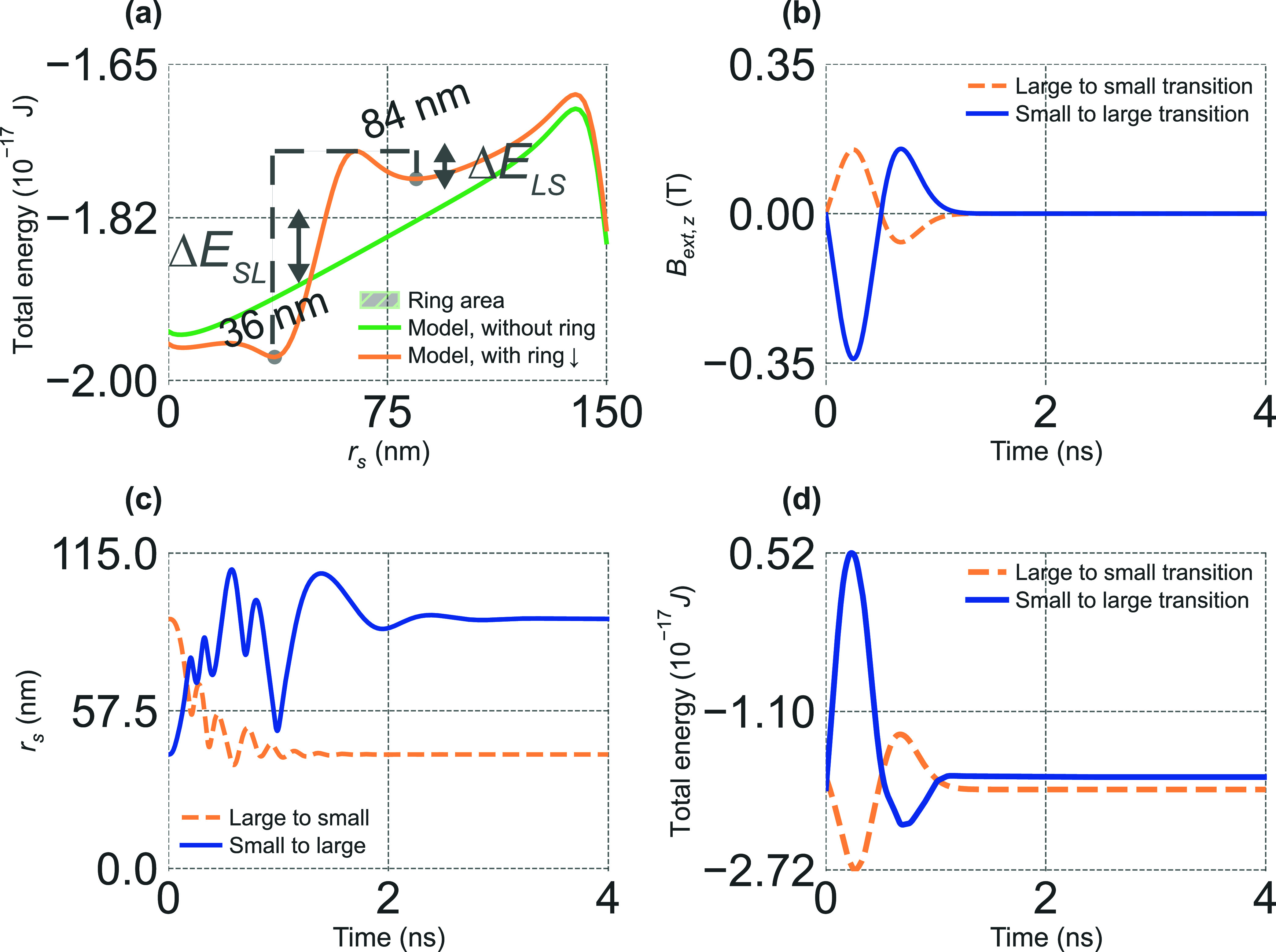
Switching between multistable skyrmion states using external magnetic field pulses. The simulations correspond to system parameters (*D* = 1.2 mJ/m^2^, *L*_d_ = 1.2 nm, *r*_d_ = 150 nm, ring *r*_in_ = 40 nm, *r*_out_ = 60 nm, *L*_r_ = 3.6 nm) resulting in stable states at approximately *r*_s_ = 36 nm (smaller radius state) and *r*_s_ = 84 nm (larger radius state). (a) Analytically calculated energy landscape showing two distinct energy minima corresponding to the smaller and larger radius skyrmion states, indicated by gray dots. The shaded region marks the radial extent of the ring. The orange line represents the calculated energy profile with the ring. (b) Applied out-of-plane external magnetic field pulses (*B*_ext,*z*_) as a function of time. The solid blue line shows the negative pulse (≈−0.34T) used to trigger the ‘smaller to larger transition’, and the dashed orange line shows the positive pulse (≈0.15T) used to trigger the ‘larger to smaller transition’. Pulses have a duration of approximately 1 ns. (c) Time evolution of the skyrmion radius (*r*_s_) during the switching processes initiated by the pulses shown in (b). The solid blue line depicts the expansion from the smaller (≈36nm) to the larger radius (≈84nm) , while the dashed orange line shows the contraction from the larger to the smaller radius. Relaxation to the stable state occurs within approximately 2.1 ns. (d) Time evolution of the total system energy during the switching processes, corresponding to the radius dynamics shown in (c). The energy rapidly changes during the pulse application and then relaxes toward the minimum energy value for the respective final state. All simulations were performed for α = 0.1.

To investigate transitions between these states, we use short external magnetic field pulses applied out-of-plane (*B*_ext,*z*_) with varying amplitudes and durations. The transition from the smaller state to the larger state requires a negative field pulse that temporarily reduces the effective field, allowing the skyrmion to expand. We found that the pulse amplitude needed for this transition is approximately −0.34 T with a duration of about 1 ns. The optimized field pulse is shown in Figure 4(b) with the solid blue line. Conversely, switching from the L to S state requires a positive field pulse (dashed orange line) of approximately 0.15 T and the same duration as that of compressing the skyrmion.

It is noteworthy that the specific temporal profiles of the magnetic field pulses shown in Figure 4(b) do not have simple shape. This tailored shaping stems from the complexity of driving the skyrmion’s size change across the energy barrier created by the ring. The pulse profile typically features an initial phase with a strong rising or falling trend to initiate the transition, followed by a phase with an opposing trend (e.g., returning toward zero or slightly overshooting). This counterphase is intentionally designed to mitigate the skyrmion’s inertial response, effectively damping the resulting oscillations (like the breathing mode) and thereby shortening the relaxation time to the new equilibrium state. The necessary pulse duration and the subsequent relaxation time are inherently dependent on the Gilbert damping parameter (α) of the nanodot. The pulse profile itself can be optimized for a specific damping value to minimize the switching time and energy. While the process warrants further optimization, our aim here was to demonstrate the feasibility of such controlled switching.

The resulting switching dynamics are detailed in Figure 4(c) and (d). Figure 4(c) shows the time evolution of the skyrmion radius (*r*_s_) during the switching processes initiated by the pulses shown in Figure 4(b). It clearly depicts the radius changing from the initial value ( ≈36nm or ≈84nm ) to the final value ( ≈84nm or ≈36nm , respectively). The process involves a rapid change in the skyrmion radius during the pulse duration, followed by damped oscillations around the new equilibrium state. Relaxation to the stable state occurs within approximately 2 ns for the parameters and pulses used here. Higher damping accelerates energy dissipation and stabilization, while lower damping prolongs oscillations and may induce unwanted harmonic behavior,^30^ although the device operation principle remains unchanged. Figure 4(d) presents the corresponding time evolution of the total system energy. The energy rapidly changes during the pulse (as the system is excited) and then relaxes toward the minimum energy value for the respective final state, also exhibiting oscillations consistent with the skyrmion radius dynamics. The total switching time, including relaxation, is approximately 1.5 to 2 ns. These findings collectively establish a comprehensive framework for controlling skyrmion states in nanodot–ring hybrid structures, paving the way for advanced spintronic devices that exploit the unique properties of skyrmion multistability for multistate memory and logic applications.^4,31^

Our study, which combines analytical modeling and micromagnetic simulations, shows that the stray field from a ferromagnetic ring with out-of-plane magnetic anisotropy can stabilize Néel skyrmions within an adjacent circular thin nanodot, even in the absence of Dzyaloshinskii–Moriya interaction. Furthermore, we show that the ring creates a complex energy landscape in which a stable skyrmion can exist with two or more different diameters. The specific stable states of skyrmions depend on the DMI of the nanodot and the inner and outer radii of the ring and can be controlled by the polarity of the magnetization of the ring. Moreover, we demonstrate that transitions between these distinct stable skyrmion states can be triggered and controlled using nanosecond external magnetic field pulses. The shape of this pulse can be designed to overcome the ring-induced energy barriers and suppress skyrmion annihilation, highlighting the feasibility of practical write operations in potential multilevel memory or logic devices, which might be challenging, but developed with current fabrication technologies.^32,33^ This compares favorably with conventional magnetic memory technologies, positioning skyrmion-based multistable memory as a competitive candidate for high-speed, high-density storage applications and potentially novel neuromorphic computing concepts, especially considering the potential for further optimization of the switching protocol.

## Supplementary Material



## Notes

Detailed derivation of the analytical model (variational approach, DeBonte ansatz, energy calculations); analysis of the ring stray field using Fourier–Bessel expansion and magnetostatic potential; description of parameters for Ir/Co/Pt nanodots and Co/Pd rings, and micromagnetic simulation Mumax3 implementation; thermal stability analysis based on the Néel–Arrhenius relation with energy barriers and corresponding lifetime estimates; assessment of ring–dot separation effects demonstrating robustness up to approximately a dozen nanometers; topological charge calculations for skyrmions; and supporting Figures S1–S5 comparing analytical and simulation results, field distributions, energy landscapes, separation effects, and magnetization configurations (PDF)
