# Supplementary material for: Beyond Fixed-Size Skyrmions in Nanodots: Switchable Multistability with Ferromagnetic Rings
Source: Nano Lett. 2025 Sep 11;25(38):13988–95. doi: 10.1021/acs.nanolett.5c02678 (PMC12464988; doi:10.1021/acs.nanolett.5c02678)
Supplement: Supplementary file 1 [file nl5c02678_si_001.pdf]

Supporting Information

Beyond fixed-size skyrmions in nanodots: switchable multistability with ferromagnetic ring

Mateusz Zelent,[*,†] Maciej Krawczyk,[‡] and Konstantin Y. Guslienko[¶,§,‖]

†*Faculty of Physics and Astronomy, Adam Mickiewicz University in Poznan, Uniwersytetu Poznańskiego 2, PL-61-614 Poznan, Poland*

‡*Faculty of Physics and Astronomy, Adam Mickiewicz University, Poznan, Uniwersytetu Poznańskiego 2, PL-61-614 Poznan, Poland*

¶*Depto. Polimeros y Materiales Avanzados: Fisica, Quimica y Tecnologia, Universidad del País Vasco, UPV/EHU, 20018 San Sebastian, Spain*

§*EHU Quantum Center, University of the Basque Country, UPV/EHU, 48940 Leioa, Spain*

‖*IKERBASQUE, the Basque Foundation for Science, 48013 Bilbao, Spain*

E-mail: mateusz.zelent@amu.edu.pl

Analytical model

To gain physical insight into the stability of Néel skyrmions within the ultrathin magnetic nanodot, complementing micromagnetic simulations, we develop an analytical model based on a variational approach. This model first establishes the energy landscape for an isolated skyrmion in the circular dot, providing a baseline upon which the effects of the external ring stray field is later assessed.

The system's geometry is illustrated in Fig. 1(a) (the main text), consisting of an Ir/Co/Pt multilayer nanodot hosting the skyrmion, with a Co/Pd ferromagnetic ring deposited on top of it with strong out-of-plane uniaxial anisotropy. The nanodot has a fixed thickness of $L_d = 1.2$ nm, and its radius r_d is varied from 100 to 200 nm. The ring's thickness L_r and its inner/outer radii also serve as tunable parameters. For the presentation of the spatial distribution of the magnetostatic field, the nanodot radius is depicted as $r_d = 100$ nm in the illustrations to enhance clarity (See Fig. 1, and Fig. S1-2). In the figures showing the energy dependence on radius, the results were obtained for a nanodot with a radius of $r_d = 200$ nm, while for skyrmion switching we used $r_d = 150$ nm.

The total magnetic energy density of our system is given by:

$$e(\mathbf{m}) = A(\nabla \mathbf{m})^2 + e_{\mathrm{DMI}}(\mathbf{m}) - K_u m_z^2 + e_m(\mathbf{m}), \tag{1}$$

where A is the exchange stiffness constant, K_u is the uniaxial anisotropy constant, e_{DMI} represents the interfacial DMI energy density, and e_m corresponds to the magnetostatic energy density.

For a thin circular magnetic dot with radius r_d and thickness L_d nm, we parameterize the magnetization using the unit vector $\mathbf{m} = \mathbf{m}(\Theta, \Phi)$, where the spherical angles Θ and Φ depend on the polar coordinate vector $\rho = (\rho, \phi)$ in the dot plane. The total energy is given by $E[\mathbf{m}] = L_d \int d^2\rho\, e(\mathbf{m})$, where the integration is performed over the nanodot area.

The DMI energy density takes the form:

$$e_{\mathrm{DMI}} = D[m_z(\nabla \cdot \mathbf{m}) - (\mathbf{m} \cdot \nabla)m_z] \tag{2}$$

where D is the interfacial DMI parameter. The m_z is the z-component of magnetization.

The magnetostatic energy is generally non-local, but within the limit of an ultrathin dot ($\beta = L_d/r_d \ll 1$), it can be simplified and expressed in local form as:

$$e_m(\mathbf{m}) = \frac{\mu_0 M_s^2 m_z^2}{2}. \tag{3}$$

Therefore, we account for the effective anisotropy via a re-normalized uniaxial anisotropy constant $K = K_u - \mu_0 M_s^2/2$. Additionally, we define the magnetic material quality factor as $Q = 2K_u/\mu_0 M_s^2$, and we consider systems where $Q \geq 1$.

For axially symmetric skyrmions, the magnetization angle Θ depends only on the radial coordinate ρ, $\Theta = \Theta(\rho)$ and $\Phi = \phi + \phi_0$, where the helicity $\phi_0 = 0$ or π for Néel skyrmions. The skyrmion topological number is given by $N = [\cos(\Theta(0)) - \cos(\Theta(r_d))]/2$. We assume $\tan[\Theta(r)/2] = \exp[-f(r)]$, $(r = \rho/l)$ and use the DeBonte ansatz[1] $f(r) = \ln\left(\frac{r}{R_s}\right) + \frac{1}{\delta}(r - R_s)$ to reproduce skyrmion profile, where we define the skyrmion radius $R_s = r_s/l$ as the distance where $m_z(r) = 0$ and $l = \sqrt{A/K}$, corresponding to $\Theta(r_s) = \pi/2$ or $f(R_s) = 0$. Parameter δ corresponds to the skyrmion wall width. The magnetization components within anzatz are $m_z(r) = \cos\Theta(r) = \tanh(f(r)$ and $m_\rho(r) = \sin\Theta(r) = \frac{1}{\cosh(f(r))}$.

A skyrmion state is defined as stable when it has the lowest energy (ground state) compared to other magnetization states, and metastable when its energy is higher than that of another magnetization configuration but separated by an energy barrier. We calculate the skyrmion energy from Eq. (1-3) to determine regions of skyrmion metastability. We get the expressions of energy versus skyrmion radius, which allows us to calculate the radially symmetric skyrmion energy as:

$$E(r_s) = 2\pi A L_d \int_0^{R_d} r \Bigg[\left(\frac{1}{r^2} + 1\right) m_\rho^2 + \left(\frac{1}{\delta} + \frac{1}{r}\right)^2 m_\rho^2 + d\left(-\left(\frac{1}{\delta} + \frac{1}{r}\right) m_\rho + \frac{1}{r} m_\rho m_z\right)\Bigg] dr - \pi A L_d R_d^2. \tag{4}$$

where the radial coordinate $r = \rho/l$, dot radius R_d and skyrmion wall width δ are in units of l.

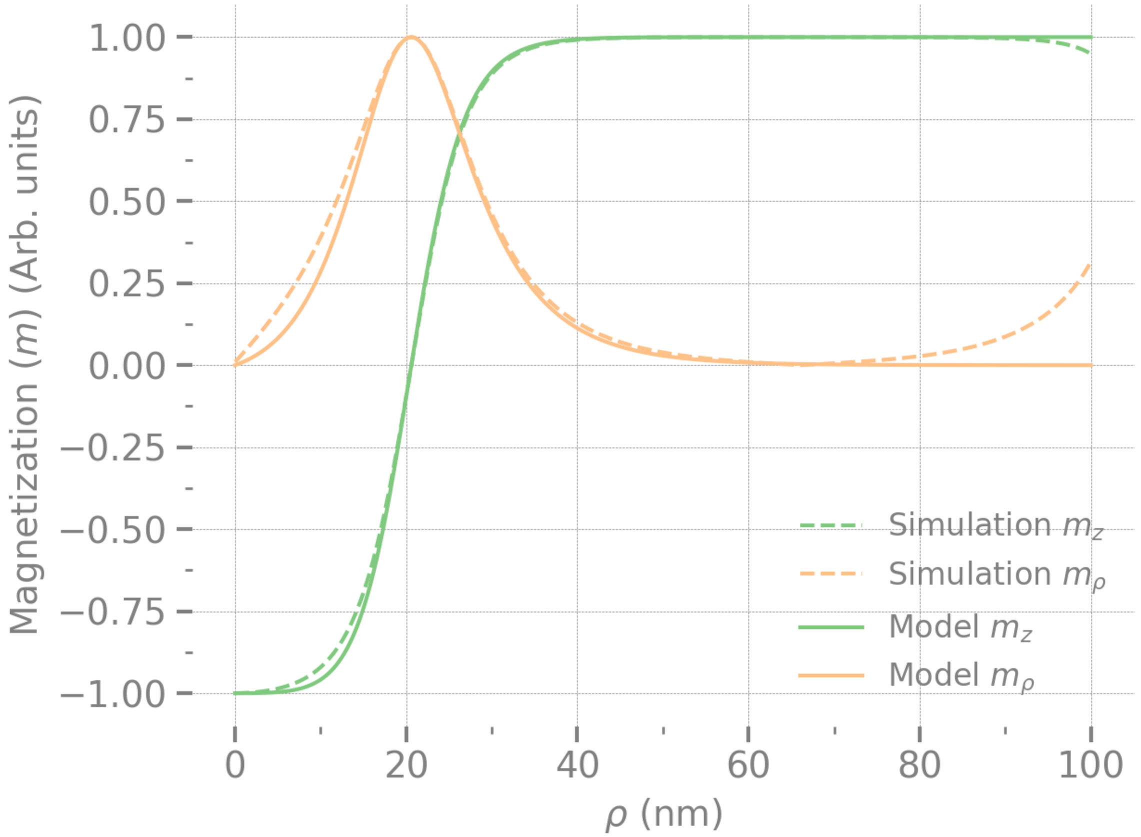

Figure S1: (a) Comparison of simulated and modeled 20 nm radius Néel type skyrmion magnetization profiles in an ultrathin multilayer nanodot. The graph shows the in-plane (orange lines) and out-of-plane (green lines) components of the magnetization as a function of the radial coordinate ρ. The dashed lines correspond to micromagnetic simulations, while the solid lines represent the analytical model based on the DeBonte ansatz.

Figure S1 compares the magnetization distribution of the skyrmion obtained using an analytical approximation with that obtained using micromagnetic simulations. The DeBonte ansatz accurately captures both the in-plane and out-of-plane magnetization components as functions of the radial coordinate, though it shows minor deviations near the edges because boundary effects (arising due to finite value of the DMI energy) are not fully accounted for in the analytical model. This model has been previously applied to isolated nanodots as reported in Refs. [2,3].

Ring stray magnetic field

The second crucial component of our model addresses the interaction between the skyrmion and the magnetostatic stray field $\mathbf{H}_{\rm ring}$ generated by the ferromagnetic ring. The Co/Pd multilayer ring with strong perpendicular magnetic anisotropy is characterized by its outer radius $r_{\rm out}$, inner radius $r_{\rm in}$ (both in reduced units), and thickness $L_{\rm r}$, serving as a local source of the magnetic field that fundamentally modifies the skyrmion energy landscape.

The stray field generated by the ring can be decomposed into two components: (a) the out-of-plane component $H_z(\rho, z)$, and (b) the in-plane radial component $H_\rho(\rho, z)$, which is non-uniform and reverses direction near the inner and outer edges of the ring.

The influence of these field components on skyrmion stability can be understood through their interaction with the skyrmion magnetization. The out-of-plane component H_z directly modifies the effective anisotropy, either enhancing or reducing it depending on the relative orientation of H_z and the skyrmion core magnetization. Meanwhile, the radial component H_ρ introduces a torque that effectively acts as an additional DMI-like contribution, influencing the skyrmion diameter and chirality. In particular, H_ρ reaches maxima near the inner and outer radii of the ring, with opposite signs, creating regions where the skyrmion wall energetically prefers to localize.

The total energy of the system with the skyrmion of radius r_s, incorporating the inter-

action with the stray field, becomes:

$$E_{\text{total}}(r_s) = E(r_s) - \mu_0 M_s \int_0^{r_d} \int_0^{L_d} 2\pi\rho \left[m_\rho(\rho) H_\rho(\rho, z) + m_z(\rho) H_z(\rho, z)\right] dz d\rho, \tag{5}$$

where the first term $E(r_s)$ is given by Eq. (4), representing the intrinsic skyrmion energy in the absence of the ring, and the second term describes the Zeeman-like interaction energy between the skyrmion magnetization and the ring-generated magnetic field.

To calculate the spatial distribution of the stray field components, we employ a semi-analytical approach based on the Fourier-Bessel expansion of the magnetostatic potential. For a uniformly magnetized ring with perpendicular magnetization $M_{s,\text{r}}$, the field components are given by:

$$H_\rho(\rho, z) = M_{s,\text{r}} \int_0^\infty J_1(k\rho) \left[J_1(kR_{\text{in}}) - J_1(kR_{\text{out}})\right] e^{-k|z|}(1 - e^{-kL_{\text{r}}}) k \, dk, \tag{6}$$

$$H_z(\rho, z) = M_{s,\text{r}} \int_0^\infty J_0(k\rho) \left[J_1(kR_{\text{in}}) - J_1(kR_{\text{out}})\right] e^{-k|z|}(1 - e^{-kL_{\text{r}}}) k \, dk, \tag{7}$$

where $J_n(x)$ are Bessel functions of the first kind, k is the wavevector in the Fourier-Bessel space, and the expressions account for the finite thickness of the ring through the factor $(1 - e^{-kL_{\text{r}}})$. The calculated spatial cross-section below the ring is shown in Fig. S2 (a,b,d – simulations, c – analytical calculations).

As demonstrated in Fig. S2(a-d), the resulting spatial distribution of these field components creates characteristic patterns that profoundly influence skyrmion stability. The H_z component forms a circular band aligned with the ring position, while the H_ρ component exhibits sharp transitions with sign changes at the inner and outer edges of the ring. These field gradients generate effective potential wells at specific radii within the nanodot, leading to the discrete stable skyrmion diameters observed in our calculations and simulations.

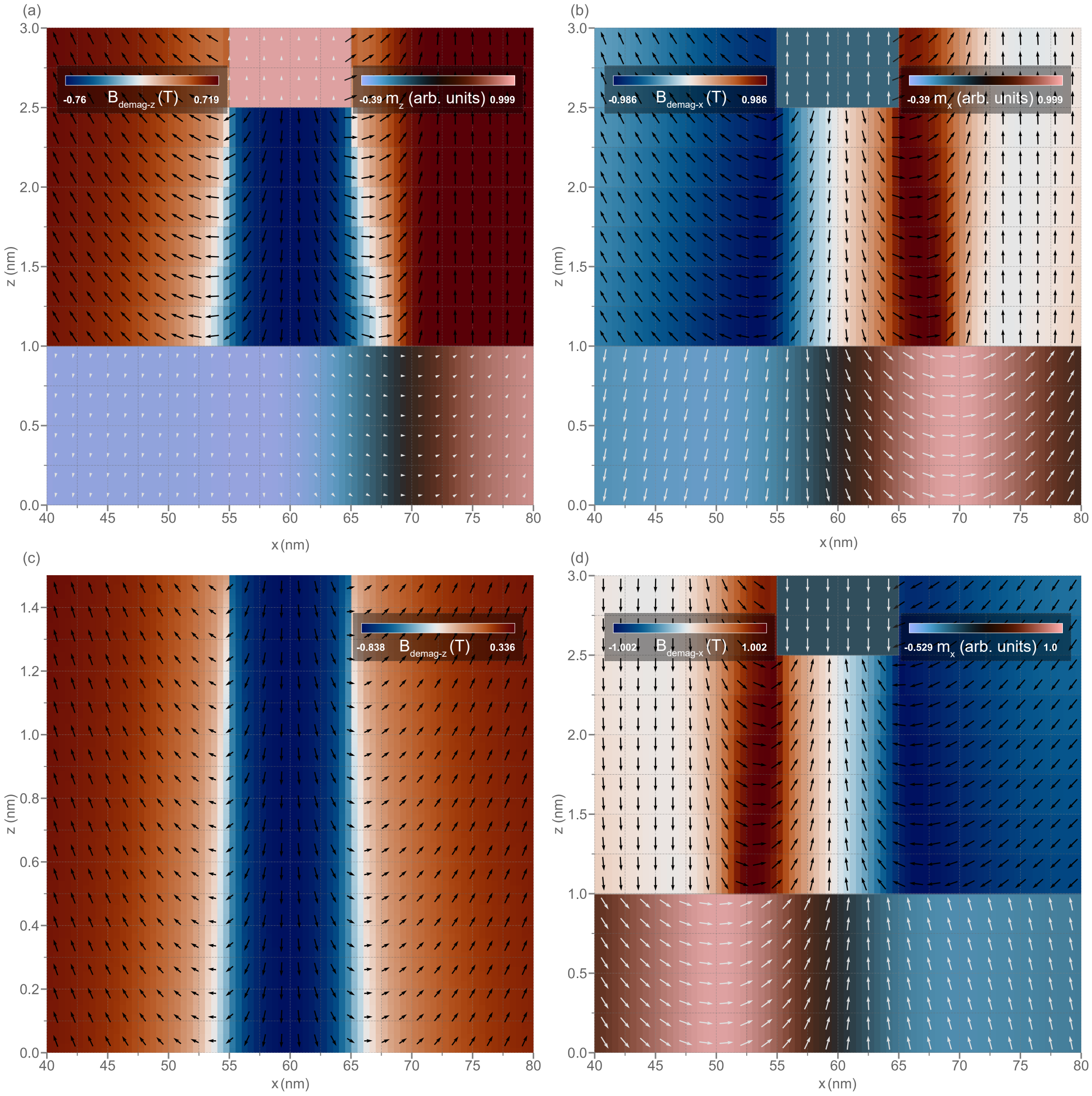

Figure S2: Spatial visualization of simulated magnetic fields (demagnetizing field $\mathbf{B}_{\text{demag}}$) and magnetization ($\mathbf{m}$) compared with an analytical calculation (B_z) in the $y-z$ cross-section for different stable skyrmion states induced by the ring. Colormaps indicate the magnitude of the specified component (see color bars). Black arrows represent field vector components (B_y, B_z or $B_{\text{demag-y}}, B_{\text{demag-z}}$), while white arrows represent magnetization vector components (m_y, m_z) in the $y-z$ plane. (a, b) Simulated state for ring magnetized upward (↑), stabilizing a large-radius skyrmion. (a) Out-of-plane components: $B_{\text{demag-z}}$ (left, colormap) and m_z (right, colormap), with corresponding field vectors (black arrows). (b) In-plane components: $B_{\text{demag-x}}$ (left) and m_x (right). (c) Analytically calculated profile of the out-of-plane component (B_z) of the ring stray field, with corresponding field vectors (black arrows). Note the different z-axis range compared to other panels. (d) Simulated state for ring magnetized downward (↓), stabilizing a small-radius skyrmion, showing in-plane components: $B_{\text{demag-x}}$ (left, colormap with black field arrows) and m_x (right, colormap with white magnetization arrows). Note the distinct visualization style for the region $z < 1.0$ nm in panels (b) and (d). The inner and outer ring radius are $r_{in} = 55.0$ nm and $r_{out} = 65.0$ nm, respectively.

The competition between the ring-induced field and the intrinsic skyrmion energetics (exchange, DMI, anisotropy) determines the final stable or metastable states. By systematically varying the ring parameters—thickness L_r, inner radius r_in, and outer radius r_out—we can engineer energy landscapes with desired stability characteristics. Furthermore, by reorienting the ring magnetization, we can dynamically modify the energy landscape, enabling switching between different stable skyrmion diameters, as demonstrated in the main text.

Materials parameters

The physical system under study is illustrated in Fig. 1 (a). The dot features a variable diameter d with a fixed thickness of 1.2 nm. We consider IrCoPt materials with the parameters taken from the literature.[4,5] We use the following material parameters for a nanodot: saturation magnetization $M_\mathrm{s} = 956$ kA/m, exchange stiffness constant $A = 10$ pJ/m, D – changes in a range from 0 to 2.6 mJ/m^2, out-of-plane magnetic anisotropy constant $K_\mathrm{u} = 0.8$ MJ/m^3 and Gilbert damping constant $\alpha = 0.1$. For ring, we assume Co/Pd with the following magnetic parameters: $M_\mathrm{s,r} = 810$ kA/m, $A = 13$ pJ/m, out-of-plane magnetic anisotropy constant $K_\mathrm{u} = 0.45$ MJ/m^3 and $\alpha = 0.1$.

Micromagnetic simulations

The micromagnetic simulations are performed by using the Mumax3[6] which solves the Landau-Lifshitz-Gilbert equation:

$$\frac{\mathrm{d}\mathbf{m}}{\mathrm{d}t} = \gamma\mu_0 \frac{1}{1+\alpha^2}(\mathbf{m} \times \mathbf{H}_\mathrm{eff}) + \alpha\mu_0 \left(\mathbf{m} \times (\mathbf{m} \times \mathbf{H}_\mathrm{eff})\right), \tag{8}$$

where $\mathbf{m} = \mathbf{M}/M_\mathrm{s}$ is the normalized magnetization, $\mathbf{H}_\mathrm{eff}$ is the effective magnetic field acting on the magnetization, $\gamma = -1.7595 \cdot 10^{11}$ Hz/T is the gyromagnetic ration. In this paper, the following components were considered for the effective field $\mathbf{H}_\mathrm{eff}$: demagnetizing field $\mathbf{H}_\mathrm{d}$, exchange field $\mathbf{H}_\mathrm{ex}$, Dzyaloshinskii-Moriya exchange field $\mathbf{H}_D$, and uniaxial anisotropy

field $\mathbf{H}_{\mathrm{Ku}}$. External magnetic field and thermal effects were neglected. Thus, the effective field $\mathbf{H}_{\mathrm{eff}}$ is expressed as:

$$\mathbf{H}_{\mathrm{eff}} = \mathbf{H}_{\mathrm{d}} + \mathbf{H}_{\mathrm{ex}} + \mathbf{H}_{\mathrm{D}} + \mathbf{H}_{\mathrm{Ku}}, \tag{9}$$

where

$$\mathbf{H}_{\mathrm{ex}} = 2\frac{A}{\mu_0 M_{\mathrm{s}}}\Delta\mathbf{m}, \tag{10}$$

and

$$\mathbf{H}_{\mathrm{D}} = \frac{2D}{\mu_0 M_{\mathrm{s}}}\left(\frac{\partial m_z}{\partial x}, \frac{\partial m_z}{\partial y}, -\frac{\partial m_x}{\partial x}, -\frac{\partial m_y}{\partial y},\right). \tag{11}$$

The uniaxial anisotropy is accounted in the form:

$$\mathbf{H}_{\mathrm{Ku}} = \frac{2K_{\mathrm{u}}}{\mu_0 M_{\mathrm{s}}}\left(\mathbf{u}\cdot\mathbf{m}\right)\mathbf{u}, \tag{12}$$

where K_{u} is the first order uniaxial magnetic anisotropy constant and $\mathbf{u}$ is a unit vector indicating the anisotropy direction.

The studied system was discretization uniformly with $0.75 \times 0.75 \times 1.2$ nm^3 unit cells to precisely imitate the rounded geometries with high accuracy. To compute the energy landscape $E(r_s)$ as a function of skyrmion radius r_s (e.g., dashed lines in Fig. 2), we employed a *frozen spins* technique adapted for micromagnetic simulations.[4] For target radius r_s, we define a narrow ring of width 1 cell size (0.75 nm) centered at the skyrmion edge. Within this ring, the magnetization is initialized such that $m_z = 0$ and the in-plane component points radially outward, i.e., $m_\rho = 1$, corresponding to a radial-vortex-like configuration at the domain wall. The magnetization inside the ring is oriented antiparallel to that outside the ring, facilitating the formation of a Néel-type skyrmion with radius r_s. Only the spins within

this narrow ring are constrained during the simulation; all other spins are free to relax. This enables the system to reach a local energy minimum for a fixed skyrmion radius without imposing any assumptions on the detailed skyrmion profile. Unlike the semi-analytical model, which requires a predefined ansatz, this method preserves full flexibility of the internal structure. Repeating the minimization over a range of radii r_s yields the full energy profile $E(r_s)$, revealing stable and metastable configurations as well as energy barriers between them.

The multi-stable skyrmion states were computed by performing simulations of skyrmion stabilization as a function of r_s, assuming the two skyrmion relaxations, one when the initial state is a large-diameter skyrmion (75% of the disk diameter) and the second with a small-diameter skyrmion (10% of the disk diameter).

The stray field analysis (Fig.1, Fig S2 a-d) involves calculating the field generated by the ring in its uniformly magnetized state. The switching simulations (Fig. 4) involves applying time-dependent external field pulses ($\mathbf{H}_{\text{ext}}(t)$) and solving the full LLG equation (8), without frozen-spins technique, to observe the dynamic evolution of the skyrmion radius and system energy.

Thermal stability

We have added quantitative lifetime estimates using the Néel-Arrhenius relation:

$$\tau = \tau_0 \exp\left(\frac{\Delta E}{k_B T}\right) \tag{13}$$

where τ_0 is the attempt time, ΔE denotes the energy barrier, defined as the difference between the total energy at the closest barrier and the corresponding energy minimum, and $k_B T = 4.1 \times 10^{-21}$ J at room temperature (300 K). The attempt time is a crucial parameter that depends on the dominant skyrmion annihilation mechanism. Based on experimental and theoretical studies,[7,8] two main regimes are identified:

- **Internal mode fluctuations:** $\tau_0 \approx 10^{-12}$ s — characterized by breathing mode

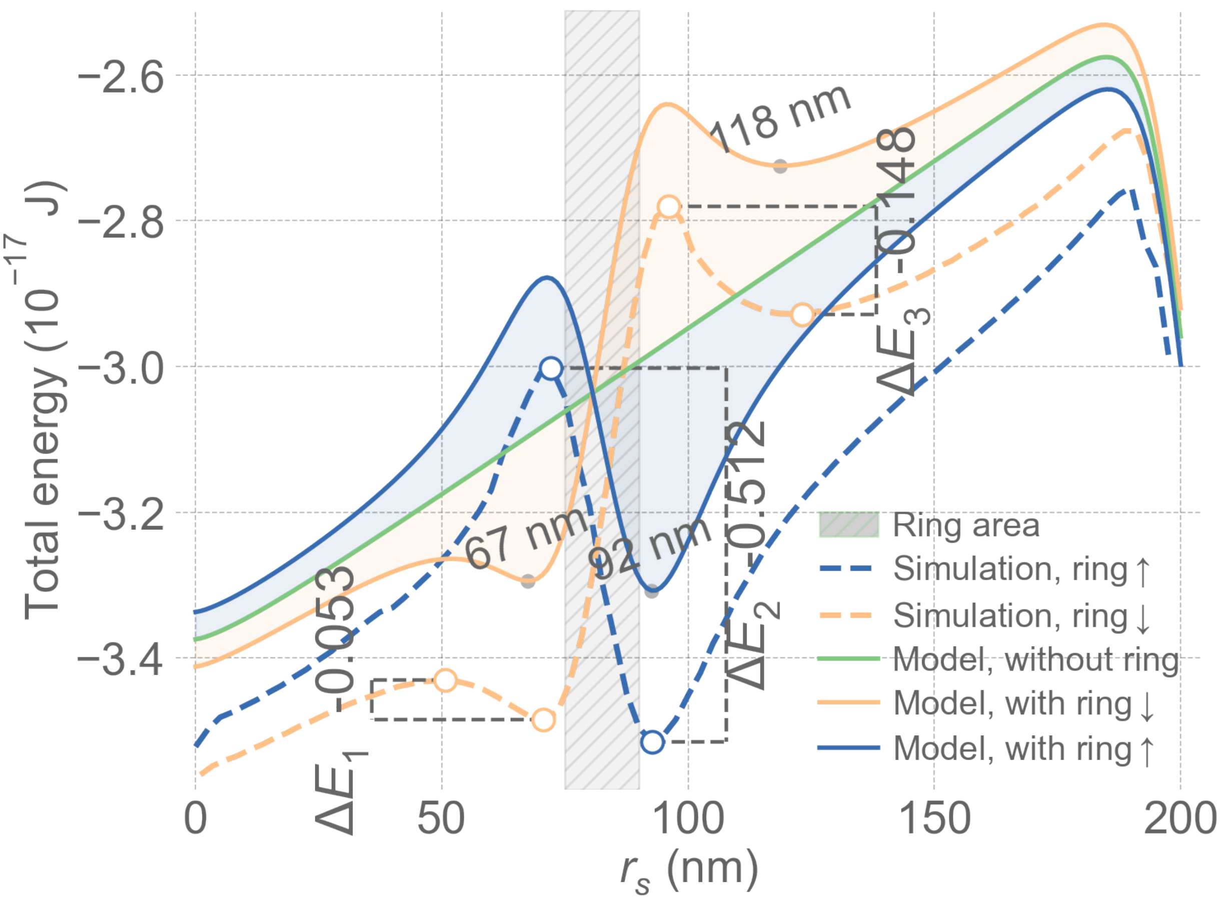

Figure S3: Total magnetic energy of the Néel skyrmion as a function of its radius r_s for negative (a) and positive (b) ring polarization for the DMI-free case taken from the main manuscript (Fig. 2). The solid lines represent the analytical model, with different colours corresponding to the given ring-dot separation (sep) values.

oscillations and radial fluctuations of the skyrmion core,

- **Boundary-mediated annihilation:** $\tau_0 \approx 10^{-9}$ s — involving skyrmion migration to sample edges or defects followed by collapse.

The choice of τ_0 significantly impacts lifetime predictions, with the boundary-mediated mechanism typically being more relevant for confined geometries like nanodots, as demonstrated in recent theoretical study.[9]

In Fig. S3 (the same as Fig. 2 in the manuscript), we select the three distinct energy barriers for both ring polarization directions. Table 1 presents the calculated lifetimes for

Table 1: Updated energy barriers and corresponding lifetimes for different switching pathways. Both limiting cases of attempt time τ_0 are shown, following Bernand-Mantel et al.[7]

**Barrier**	**Energy (J)**	$\Delta E/k_BT$	**Lifetime** ($\tau_0 = 10^{-12}$ **s**)	**Lifetime** ($\tau_0 = 10^{-9}$ **s**)
ΔE_1	0.053×10^{-17}	129	2.1×10^{44} s ($6.7e^{36}$ years)	2.1×10^{47} s (6.7×10^{39} years)
ΔE_2	$0.148e^{-17}$	361	2.1×10^{145} s	2.1×10^{148} s
ΔE_3	$0.512e^{-17}$	1249	2.1×10^{530} s	2.1×10^{533} s

these barriers considering both limiting cases of the attempt time. The calculated energy barriers (129-1249 k_BT) yield astronomically large lifetimes. In fact, this may indicate that our theoretical barriers may overestimate the actual thermal stability. This discrepancy suggests several important considerations. In particular, our theoretical model may not fully capture all dissipation mechanisms present in real devices, including edge and bulk defects.

However, for realistic device implementation, energy barriers in the range of 50-100 k_BT, would provide enough stable lifetimes of decades to centuries (well exceeding the > 10 year requirement for non-volatile memory) while enabling feasible switching with applied fields of reasonable magnitude. Thus, further work is required to minimize the energy cost of the switching preserving sufficient skyrmion stability.

Influence of ring-dot spacer thickness on skyrmion stability

The potential barriers decrease as the separation increases, due to the weakened magnetostatic stray field from the ring. As shown in Fig. S4 for both ring magnetisation polarisations, a separation of up to a dozen nm preserves the valuable energy minimum. It is worth noting that the reduction in field intensity caused by increased separation can be compensated for by increasing the ring thickness, which provides design flexibility for experimental implementation.

Topological properties

The skyrmion topological charge (skyrmion number or winding number) is defined as:

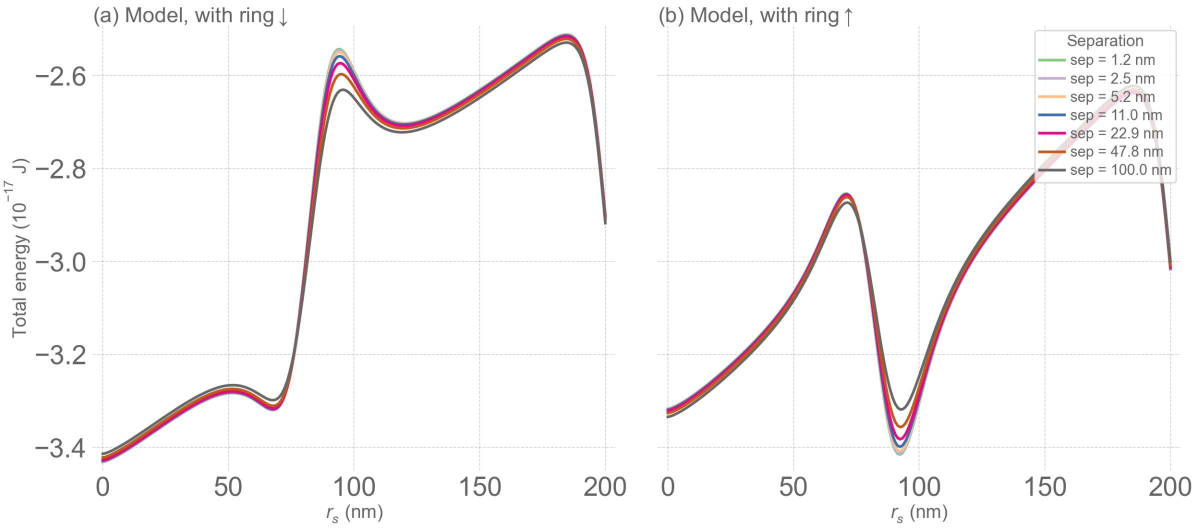

Figure S4: Total magnetic energy of the Néel skyrmion as a function of its radius r_s for negative (a) and positive (b) ring polarization for DMI-free case taken from the main manuscript (Fig. 2). The solid lines represent the analytical model, with different colours corresponding to the given ring-dot separation (sep) values.

$$N_{\mathrm{sk}} = \frac{1}{4\pi} \iint \mathbf{m} \cdot \left(\frac{\partial \mathbf{m}}{\partial x} \times \frac{\partial \mathbf{m}}{\partial y} \right) dx\, dy \tag{14}$$

where $\mathbf{m}$ is the normalized magnetization vector. This counts how many times the direction of a spin wraps around a unit sphere. For numerical calculations on a discrete 2D grid, this continuous integral is approximated using the discrete formulation:

$$N_{\mathrm{sk}} = -\frac{1}{16\pi} \sum_{i,j} \mathbf{m}_{i,j} \cdot [(\mathbf{m}_{i+1,j} \times \mathbf{m}_{i,j+1}) + (\mathbf{m}_{i-1,j} \times \mathbf{m}_{i,j-1}) - (\mathbf{m}_{i-1,j} \times \mathbf{m}_{i,j+1}) - (\mathbf{m}_{i+1,j} \times \mathbf{m}_{i,j-1})] \tag{15}$$

where $\mathbf{m}_{i,j}$ represents the normalized magnetization vector at the grid point (i, j), and the summation is performed over all grid points in the dot.

Despite the absence of DMI, the ring-stabilized skyrmion (Fig. S5a) exhibits the same topological number as conventional DMI-stabilized skyrmions, i.e.., $N_{\mathrm{sk}} = -0.99$. For the skyrmions in nanodots with DMI, there is a slight deviation from the ideal value $N_{\mathrm{sk}} =$

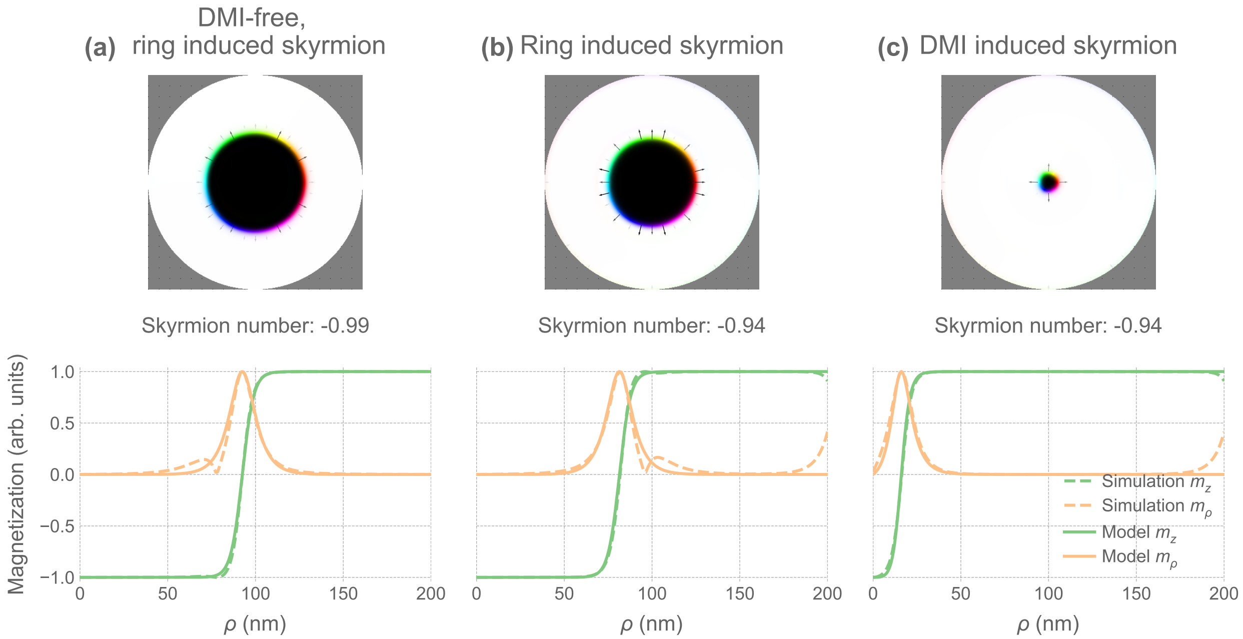

Figure S5: Comparison of skyrmion magnetization configurations showing: (a) DMI-free skyrmion with ring-induced stabilization (92 nm diameter, skyrmion number: -0.94), (b) DMI-stabilized skyrmion with ring (80 nm diameter, DMI = 1.8 mJ/m^2, skyrmion number: -0.94), and (c) DMI-stabilized (DMI = 1.8 mJ/m^2) skyrmion without ring (16 nm diameter, skyrmion number: -0.94). The color coding represents the out-of-plane magnetization component (m_z), and the arrows show the in-plane magnetization direction.

-1, e.g., $N_{\mathrm{sk}} = -0.94$ in Fig. S5(b)-(c), which is due to finite-size effects and boundary conditions (i.e., spin deflection around the dot edge). Nevertheless, the skyrmion numbers are comparable across all non-zero DMI configurations, and the same with and without the ring.

References

(1) Debonte, W. J. Properties of thick-walled cylindrical magnetic domains in uniaxial platelets. *Journal of Applied Physics* **1973**, *44*, 1793–1797.

(2) Aranda, A. R.; Guslienko, K. Y. Single Chiral Skyrmions in Ultrathin Magnetic Films. *Materials 2018, Vol. 11, Page 2238* **2018**, *11*, 2238.

(3) Tejo, F.; Riveros, A.; Escrig, J.; Guslienko, K. Y.; Chubykalo-Fesenko, O. Distinct magnetic field dependence of Néel skyrmion sizes in ultrathin nanodots. *Scientific Reports* **2018**, *8*, 6280.

(4) Zelent, M.; Tóbik, J.; Krawczyk, M.; Guslienko, K. Y.; Mruczkiewicz, M. Bi-Stability of magnetic skyrmions in ultrathin multilayer nanodots induced by magnetostatic interaction. *physica status solidi (RRL) - Rapid Research Letters* **2017**, *11*, 1700259.

(5) Moreau-Luchaire, C. et al. Additive interfacial chiral interaction in multilayers for stabilization of small individual skyrmions at room temperature. *Nature Nanotechnology* **2016**, *11*, 444–448.

(6) Leliaert, J.; Dvornik, M.; Mulkers, J.; De Clercq, J.; Milošević, M. V.; Van Waeyenberge, B. Fast micromagnetic simulations on GPU—recent advances made with Mumax3. *Journal of Physics D: Applied Physics* **2018**, *51*, 123002.

(7) Bernand-Mantel, A.; Camosi, L.; Wartelle, A.; Rougemaille, N.; Darques, M.; Ranno, L. The skyrmion-bubble transition in a ferromagnetic thin film. *SciPost Physics* **2018**, *4*, 027.

(8) Rohart, S.; Thiaville, A. Skyrmion confinement in ultrathin film nanostructures in the presence of Dzyaloshinskii-Moriya interaction. *Physical Review B* **2013**, *88*, 184422.

(9) Riveros, A.; Tejo, F.; Escrig, J.; Guslienko, K. Y.; Chubykalo-Fesenko, O. Field-

Dependent Energy Barriers of Magnetic Néel Skyrmions in Ultrathin Circular Nanodots. *Physical Review Applied* **2021**, *16*, 014068.
